# Incidentally discovered asymptomatic extrasinusal mucocele of the sphenoid greater wing: A case report and review of differential diagnoses

**DOI:** 10.1016/j.radcr.2026.03.031

**Published:** 2026-04-20

**Authors:** Achraf Amine Sbai, Mohamed Bouallou, Drissia Benfadil, Azeddine Lachkar

**Affiliations:** a Faculty of Medicine and Pharmacy, Mohammed First University, Oujda, Morocco; b Department of Otorhinolaryngology, Mohammed VI University Hospital, Oujda, Morocco; c Laboratory of Oto-Neuro-Ophthalmology (LORNO), Faculty of Medicine and Pharmacy, Mohammed First University, Oujda, Morocco; d Faculty of Medicine and Pharmacy, LAMCESM, Mohammed First University, Oujda, Morocco

**Keywords:** Extrasinusal mucocele, MRI, CT scan, Differential diagnosis, Incidental finding

## Abstract

Extrasinusal mucoceles are exceptionally rare cystic lesions that arise outside the paranasal sinuses and may pose diagnostic challenges, particularly when incidentally detected in atypical locations. Their imaging appearance can overlap with other cystic skull-base and intracranial lesions. We report the case of a 45-year-old woman with an asymptomatic extrasinusal mucocele incidentally discovered on CT performed during follow-up of a peritonsillar abscess. Imaging revealed a well-defined expansile cystic lesion in the spheno-temporo-orbital region, causing cortical thinning without surrounding intracranial edema or diffusion restriction. MRI demonstrated homogeneous fluid signal intensity, thin peripheral enhancement, and absence of solid components. Based on these radiological findings, an imaging-based diagnosis of a probable extrasinusal mucocele was favored. As the patient declined surgical intervention, a conservative approach with regular clinical follow-up every 3 months and annual MRI surveillance was adopted. At the most recent follow-up, the lesion remained radiologically stable without interval growth or new symptoms. This case underscores the importance of considering extrasinusal mucocele in the differential diagnosis of skull-base cystic lesions.

## Introduction

Paranasal sinus mucoceles are epithelium-lined cystic lesions resulting from obstruction of the normal sinus drainage pathways, leading to progressive accumulation of sterile mucus and gradual expansion of the affected sinus cavity. Although histologically benign, mucoceles may exhibit locally aggressive behavior, causing progressive bone remodeling and erosion, with displacement of adjacent anatomical structures. They most commonly involve the frontal and ethmoid sinuses, whereas sphenoidal involvement is less frequent [1].

In contrast, extrasinusal mucoceles represent an exceptionally rare entity. Unlike conventional mucoceles, these lesions develop in ectopic locations without demonstrable communication with the sinonasal cavities, and only isolated cases have been reported in the literature [2,3]. Most previously described extrasinusal mucoceles have been located in the orbit, while intracranial presentations remain extremely uncommon. The pathogenesis of these lesions is not fully understood but is thought to involve ectopic mucosal remnants of embryological origin or mucosal displacement following trauma or inflammation.

The diagnosis of extrasinusal mucoceles may be particularly challenging, especially when lesions are discovered incidentally or occur in atypical locations. Radiological evaluation plays a central role in characterization. However, the imaging appearance may overlap with a broad spectrum of cystic skull-base and intracranial lesions. The main differential diagnoses include arachnoid cysts, epidermoid and dermoid cysts, cystic neoplasms, and, less commonly, abscesses or other inflammatory lesions [4]. Careful analysis of imaging features is therefore essential to establish an accurate diagnosis and guide management.

We present a rare case of an asymptomatic extrasinusal mucocele incidentally discovered in the spheno-temporo-orbital region, emphasizing its radiological characteristics, diagnostic challenges, management considerations, and a review of previously reported cases.

## Case presentation

A 45-year-old woman was referred to our Department of Otorhinolaryngology for evaluation of an incidentally detected extrasinusal mucocele identified on imaging. The lesion was discovered during a contrast-enhanced CT scan performed for follow-up of a right peritonsillar abscess, which had resolved after antibiotic therapy with amoxicillin–clavulanic acid for 14 days.

Her medical history was significant for obsessive–compulsive disorder, treated with antidepressant medication. She had no history of chronic rhinosinusitis, prior sinonasal surgery, or craniofacial trauma.

At presentation, the patient was asymptomatic and denied headaches, nasal obstruction, rhinorrhea, visual disturbances, or neurological complaints.

Physical examination revealed a patient in good general condition, hemodynamically and respiratory stable. Nasal endoscopy showed normal nasal mucosa with patent nasal cavities and middle meatuses, and a normal nasopharynx. Cervical examination revealed no palpable lymphadenopathy. A complete neurological examination, including cranial nerve assessment, was normal. Ophthalmological examination showed no abnormalities. Ophthalmological examination was unremarkable, with normal visual acuity, preserved ocular motility, and no evidence of proptosis, diplopia, or visual field defects.

Routine laboratory investigations were performed and showed no significant abnormalities. Complete blood count, C-reactive protein, and erythrocyte sedimentation rate were within normal limits. Serum electrolytes, renal function, and liver function tests were also unremarkable.

**Fig. 1:**
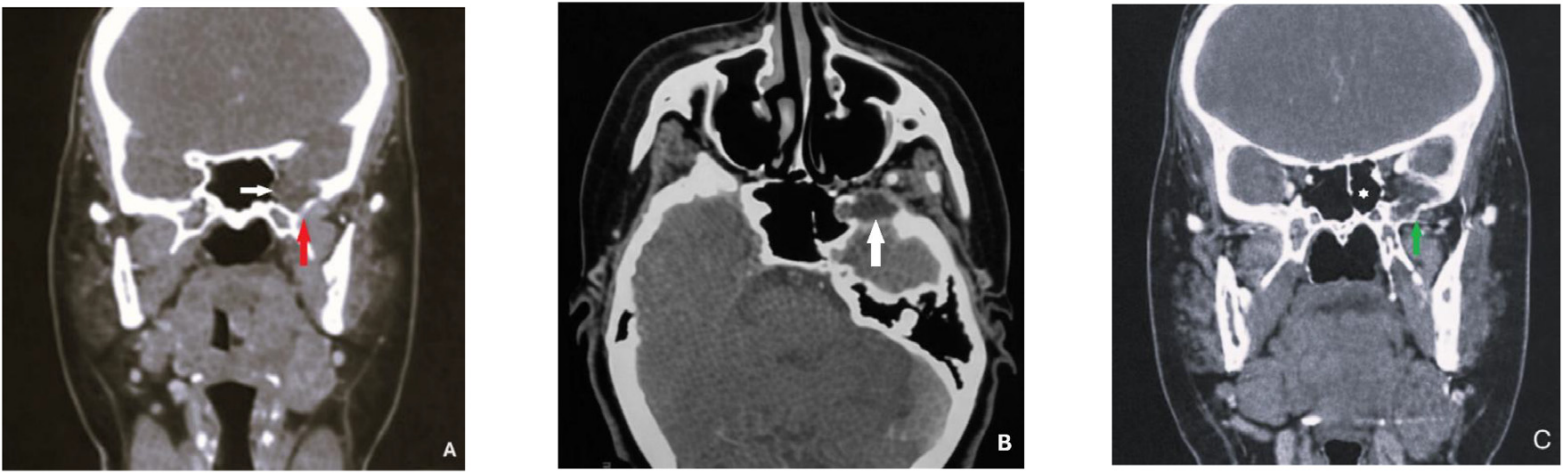
Contrast-enhanced CT of the head and neck in the parenchymal window, coronal (A) and axial (B) images, demonstrating a well-defined, smoothly marginated cystic lesion centered in the left spheno-temporo-orbital region (white arrow), extending toward the left infratemporal fossa and associated with osteolysis of the greater wing of the sphenoid (red arrow). (C) Coronal CT image in parenchymal window demonstrating well-aerated sphenoid sinuses (white star), with no communication between the mucocele and the sphenoid sinus, associated with osteolysis of the left greater wing of the sphenoid (green arrow).

A contrast-enhanced CT scan of the head and neck demonstrated a well-marginated expansile osteolytic cystic lesion centered in the left greater wing of the sphenoid, corresponding to the spheno-temporo-orbital region. The lesion causing thinning and outward bowing of the surrounding cortical bone and was located along the lateral orbital wall and the floor of the middle cranial fossa, with inferior extension toward the infratemporal fossa. The lesion exhibited fluid attenuation and no internal enhancement following contrast administration. The lesion measured approximately 24 × 11 mm in maximal axial dimensions (Fig. 1). Importantly, the lesion was in close contact with the sphenoid bone and adjacent skull-base structures, without evidence of true intracranial extra-axial extension or invasion of the adjacent brain parenchyma.

**Fig. 2:**
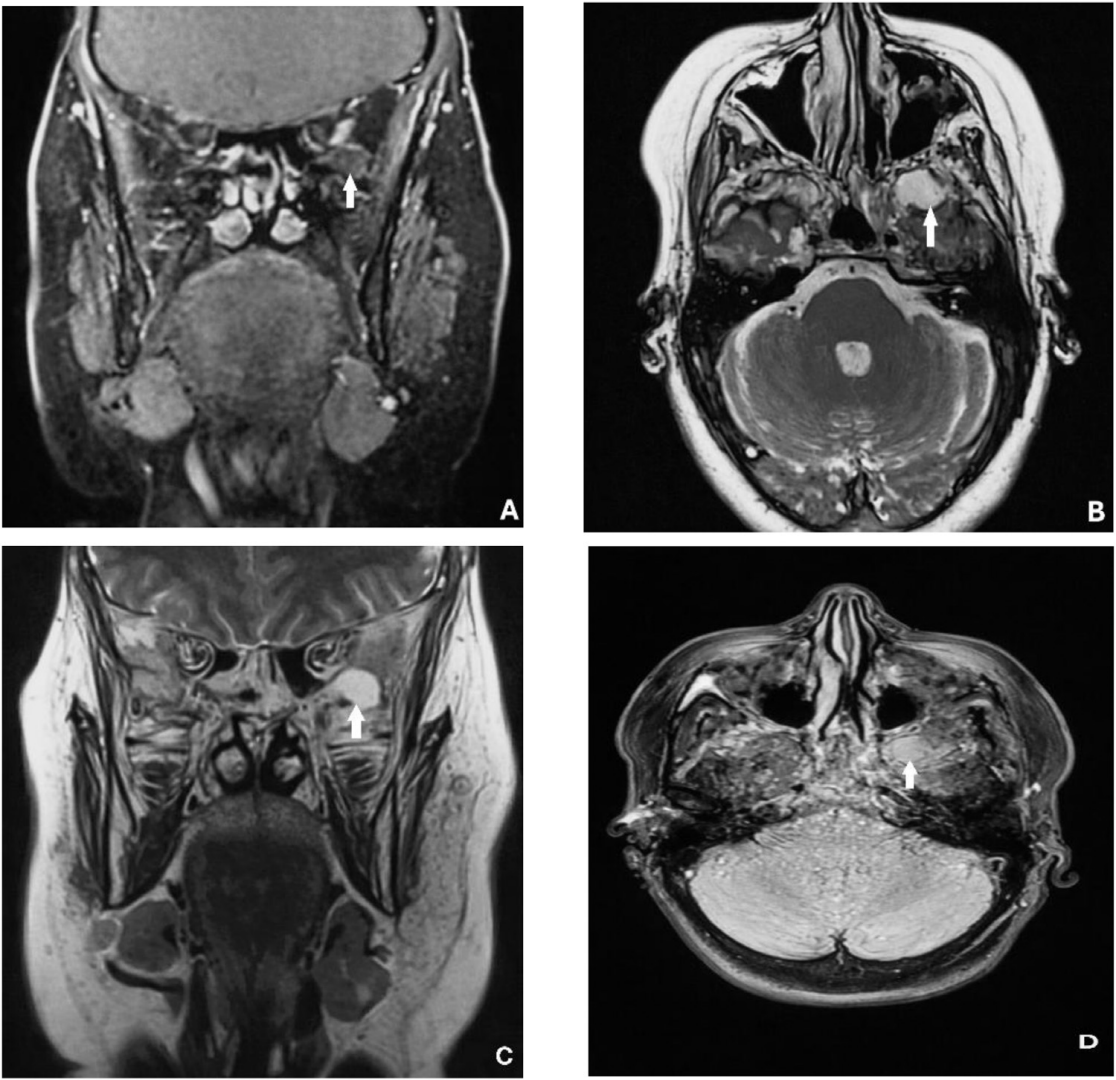
MRI findings. (A) Coronal contrast-enhanced fat-suppressed T1-weighted image showing a well-defined cystic lesion centered in the left greater wing of the sphenoid, appearing hypointense, without internal enhancement (white arrow). (B, C) Axial (B) and coronal (C) T2-weighted images demonstrating marked hyperintensity of the lesion with extension toward the left infratemporal fossa (white arrow). (D) Axial T2-FLAIR image confirming the hyperintense signal of the cystic lesion (white arrow).

**Fig. 3:**
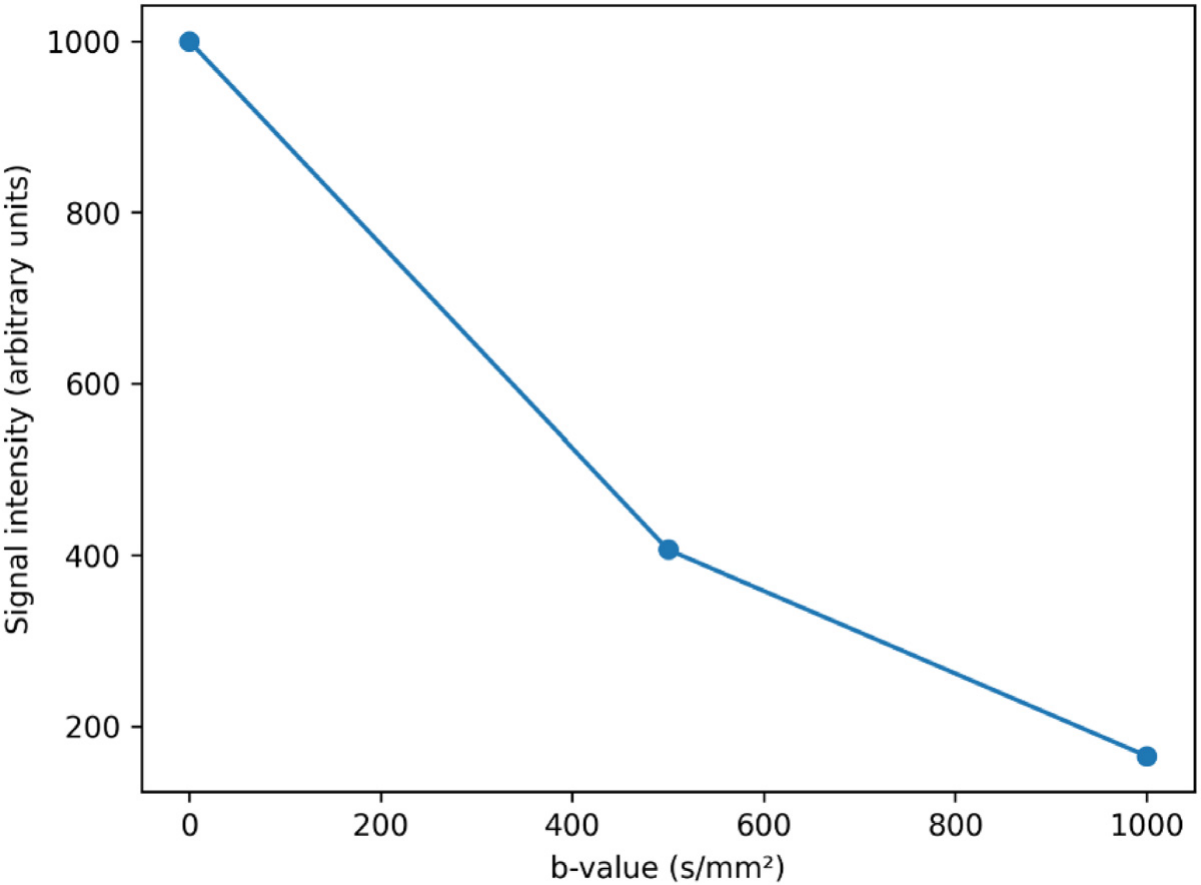
Apparent diffusion coefficient (ADC) signal decay curve in our patient confirming the absence of diffusion restriction.

Subsequent MRI confirmed a well-circumscribed lesion centered in the left greater wing of the sphenoid, with smooth margins and expansion of the surrounding bone. The MRI protocol included T1-weighted sequences before and after gadolinium administration, contrast-enhanced fat-suppressed T1-weighted sequences, T2-weighted sequences, T2-FLAIR, and diffusion-weighted imaging (DWI). The lesion appeared hypointense on T1-weighted images and hyperintense on T2-weighted images. Following gadolinium administration, a thin, regular peripheral enhancement was observed. On T2-FLAIR sequences, the lesion remained hyperintense, suggesting the presence of relatively proteinaceous fluid content rather than purely simple fluid (Fig. 2). Diffusion-weighted imaging showed no diffusion restriction on DWI with corresponding high ADC values, arguing against an epidermoid cyst or abscess (Fig. 3). No susceptibility artifacts suggestive of hemorrhage or calcification were identified on susceptibility-sensitive sequences. The adjacent brain parenchyma was normal, with no evidence of edema and mass effect.

Based on the combined clinical and imaging findings, an imaging-based diagnosis of extrasinusal mucocele was considered the most likely explanation.

Endoscopic marsupialization was recommended. However, the patient declined surgical treatment after discussion of potential risks and benefits. A conservative management strategy was therefore adopted, consisting of regular clinical follow-up every three months and MRI surveillance every 12 months.

## Discussion

Paranasal sinus mucoceles result from obstruction of the normal sinus drainage pathways, leading to progressive retention of sterile mucoid secretions within an epithelial-lined cavity and gradual expansion of the involved sinus. These lesions typically originate within a paranasal sinus and enlarge slowly, causing remodeling and erosion of adjacent bony walls with subsequent displacement of neighboring anatomical structures [5]. Mucoceles most commonly affect the ethmoid and frontal sinuses.

Progressive accumulation of mucus associated with chronic obstruction generates sustained mechanical pressure on the sinus walls, leading to gradual bone thinning and resorption, thereby allowing expansion toward adjacent orbital or intracranial structures [6]. In parallel, persistent inflammation plays a key role in this process. Inflammatory mediators, particularly prostaglandin E₂, have been identified as major contributors to the osteolytic and locally destructive behavior of mucoceles, through stimulation of osteoclast-mediated bone resorption [7].

The etiology of paranasal sinus mucoceles is multifactorial, with obstruction of the sinus ostium, often due to polyps or chronic inflammation, representing the primary initiating event [8]. Additional contributing factors include chronic sinusitis, prior facial trauma, previous sinonasal surgery, allergies, and anatomical variations that compromise normal sinus drainage [9]. Remarkably, in our case, the patient was entirely asymptomatic and presented with none of these recognized predisposing factors, supporting an idiopathic pathogenesis of this extrasinusal mucocele.

Extrasinusal mucoceles represent an exceptionally rare presentation, with only a limited number of cases reported in the literature since the first description published in 2003 [2]. Unlike conventional paranasal sinus mucoceles, which originate within a sinus cavity and gradually enlarge as a result of ostial obstruction, these lesions arise in ectopic locations without demonstrable communication with the sinonasal cavities, which may make diagnosis more challenging.

To our knowledge, isolated extrasinusal mucoceles reported in the literature have mainly been described in orbital locations. In contrast, the present case represents an exceptional intracranial presentation without sinus communication. A plausible explanation is that extrasinusal mucoceles develop from ectopic mucosal remnants of embryological origin situated outside the sinonasal cavities, which gradually enlarge due to mucus retention. Previously reported cases of extrasinusal mucoceles and their main clinical and imaging features are summarized in Table 1.

**Table 1:** Previously reported cases of extrasinusal mucoceles.

Study	Location	Symptoms	Imaging characteristics	Management
Asamoto et al. [2]	Orbit	Orbital symptoms	CT: well-defined cystic intraorbital lesion causing bone remodeling. MRI: fluid signal intensity, no solid component reported. Size not clearly specified.	Surgical treatment
Prendes et al. [3]	Orbit	Orbital symptoms	CT: well-circumscribed cystic orbital lesions without sinus communication. MRI: T1 hypointense, T2 hyperintense lesions with peripheral enhancement. Size variable depending on cases.	Surgical treatment
Alouda et al. [10]	Subcutaneous	Local swelling	CT: well-defined cystic subcutaneous lesion without bony destruction. MRI: fluid signal intensity, thin peripheral enhancement. Size approximately 3 cm (reported case).	Surgical treatment
Cancio et al. [11]	Orbital apex	Visual and orbital symptoms	CT: expansile cystic lesion at orbital apex. MRI: T1 hypointense, T2 hyperintense, peripheral enhancement; no diffusion restriction reported. Size around 2 cm in greatest dimension.	Surgical treatment
This case	Intracranial	Asymptomatic	CT: expansile osteolytic cystic lesion causing cortical thinning, fluid attenuation, no internal enhancement, measuring 24 × 11 mm. MRI: well-circumscribed lesion, T1 hypointense, T2 hyperintense, no diffusion restriction, thin peripheral enhancement	Conservative

Most intracranial mucoceles, whether of intrasinus or extrasinusal origin, are typically symptomatic at the time of diagnosis, owing to their mass effect on adjacent neural and vascular structures [12]. Reported clinical manifestations commonly include headaches, visual disturbances, cranial nerve deficits, seizures, or signs of raised intracranial pressure, which usually prompt early imaging evaluation [13]. In contrast, our case is distinguished by the complete absence of neurological or sinonasal symptoms, despite intracranial extension, with the lesion being discovered incidentally on imaging.

Imaging plays a key role in the diagnosis and management of mucoceles. CT is generally considered the first-line modality because of its excellent depiction of bony anatomy, allowing detection of sinus opacification, expansile remodeling, cortical thinning, bone erosion, and anatomical variations relevant to surgical planning. MRI provides complementary information by offering superior soft-tissue contrast, enabling better characterization of lesion content, differentiation from neoplastic or inflammatory cystic lesions, and assessment of adjacent brain parenchyma when intracranial involvement is suspected. On MRI, mucoceles typically show variable T1 signal intensity, high T2 signal intensity, and thin peripheral enhancement after contrast administration [14].

The radiological differential diagnosis of a cystic lesion located in the greater wing of the sphenoid and skull-base bone is relatively broad and includes several benign cystic entities as well as neoplastic processes. When the lesion is intraosseous and centered within the sphenoid wing, the differential diagnosis should primarily include other skull-base intraosseous or epidural cystic lesions rather than intracranial cysts in general.

Among these, arachnoid cysts represent one of the most important considerations. Arachnoid cysts are typically extra-axial lesions that follow cerebrospinal fluid signal intensity on all MRI sequences, demonstrate no enhancement, and do not cause bone erosion or remodeling in most cases [4]. Furthermore, arachnoid cysts are located within the subarachnoid space rather than within bone. In contrast, the lesion in our patient was centered within the spheno-temporo-orbital bone with associated cortical thinning and osteolysis of the greater wing of the sphenoid, indicating an intraosseous origin rather than an extra-axial CSF-containing cyst.

Dermoid cysts should also be considered among skull-base cystic lesions. These lesions often contain fat components derived from ectodermal elements, resulting in high signal intensity on T1-weighted images with signal suppression on fat-suppressed sequences. In addition, dermoid cysts are typically located along midline embryologic fusion planes and rarely produce significant expansile bone remodeling [15]. None of these imaging characteristics were observed in our patient, making this diagnosis unlikely. Moreover, dermoid cysts are most frequently reported in the frontal, parietal, or occipital regions, and involvement of the middle cranial fossa or sphenoid wing is extremely uncommon [16].

Another important consideration for a cystic lesion of the sphenoid wing is an intraosseous epidermoid cyst. Epidermoid cysts can mimic mucoceles on conventional MRI sequences, typically appearing hypointense on T1-weighted images and hyperintense on T2-weighted images. However, epidermoid cysts characteristically demonstrate marked diffusion restriction on DWI due to the presence of keratinaceous debris, a feature that was absent in our case [17]. The absence of diffusion restriction with high ADC values strongly argues against this diagnosis.

Other intraosseous skull-base lesions that may present with cystic imaging features include aneurysmal bone cysts, intraosseous meningiomas with cystic degeneration, and metastatic lesions involving the sphenoid bone.

Aneurysmal bone cysts usually demonstrate multiloculated expansile lesions with internal septations and fluid–fluid levels on MRI, findings that were not observed in our case. Intraosseous meningiomas or cystic meningiomas generally demonstrate solid enhancing components or associated dural thickening, which were absent in the present lesion. Similarly, metastatic lesions typically occur in patients with a known primary malignancy and frequently show aggressive bone destruction, irregular margins, and soft-tissue components, none of which were present in our patient [18].

Another potential differential diagnosis is a mucous retention cyst arising from the lateral recess of the sphenoid sinus, which represents a pneumatized extension of the sinus that may extend into the greater wing of the sphenoid and mimic an intraosseous lesion on imaging [19]. However, in the present case, CT demonstrated a well-aerated sphenoid sinus without visible communication with the lesion, which appeared centered within the sphenoid bone, making this diagnosis less likely. In the present case, careful evaluation of contrast-enhanced CT images demonstrated a well-aerated sphenoid sinus with intact sinus walls and no identifiable communication between the lesion and the sphenoid sinus, strongly arguing against a retention cyst arising from the lateral recess.

Finally, an abscess or inflammatory cystic process may occasionally mimic a mucocele. However, intracranial or skull-base abscesses typically demonstrate marked diffusion restriction, surrounding edema, and clinical signs of infection, features that were not present in this case.

Taken together, the combination of an intraosseous expansile lesion centered in the greater wing of the sphenoid, smooth cortical remodeling, absence of solid enhancing components, lack of diffusion restriction, and thin peripheral enhancement strongly favored the diagnosis of a mucocele over other cystic skull-base lesions. However, most reported cases were located in the orbit and were symptomatic, often presenting with proptosis or visual disturbances, which facilitated diagnosis. In contrast, our case was entirely asymptomatic and discovered incidentally, further increasing the diagnostic challenge and emphasizing the importance of careful radiological evaluation.

Despite their benign histology, mucoceles can cause serious complications due to mass effect on adjacent neural and vascular structures. Progressive expansion may result in bone thinning and remodeling, potentially compressing critical structures. In our case, the mucocele is located in close proximity to the optic nerve, carotid canal, and cavernous sinus, raising concern for ocular and neurovascular compromise. As reported by Makihara et al. [20], ocular complications, including visual disturbances, proptosis, and diplopia, are the most frequent manifestations of paranasal mucoceles. Other potential complications include cranial nerve deficits and, in rare cases, vascular involvement such as cavernous sinus thrombosis, particularly with sphenoidal mucoceles [21].

Given these risks, close clinical and radiological follow-up is essential, especially for asymptomatic patients. In the present case, surveillance with nasal endoscopy and ophthalmological examination every three months and MRI every 12 months has been adopted to detect lesion progression early and intervene before irreversible ocular or neurological sequelae occur.

Although formal guidelines are lacking for conservatively managed asymptomatic mucoceles, recent evidence supports regular clinical and radiological monitoring. In a retrospective cohort of incidentally identified paranasal sinus mucoceles, asymptomatic lesions generally demonstrated a benign clinical course, with the recommendation of interval CT or MRI at approximately 12 months to identify early changes suggestive of progression [22]. Lesions demonstrating growth or new imaging features may require shorter surveillance intervals or surgical intervention to prevent complications such as orbital or intracranial extension.

Endoscopic marsupialization remains the standard treatment for symptomatic mucoceles or those demonstrating progressive expansion, as it effectively decompresses the lesion and prevents complications, with a low recurrence rate and minimal morbidity [23]. However, lesions in technically challenging locations, such as those with extensive lateral extension or in proximity to critical neurovascular structures, may require alternative or combined approaches. In such cases, endoscopic endonasal transmaxillary routes have been reported as effective techniques to achieve adequate access and marsupialization while minimizing morbidity, providing a wide surgical field and facilitating complete drainage [24]. Given the proximity of the lesion to the optic nerve, carotid canal, and its extension into the infratemporal fossa, a transmaxillary approach was considered to provide improved access and enable safe decompression should surgical intervention become necessary.

Importantly, the present case has certain limitations. Although the imaging findings strongly suggest an extrasinusal mucocele, the diagnosis remains imaging-based, as no histopathological confirmation was obtained. Surgical biopsy or excision was initially proposed to establish a definitive diagnosis and provide treatment. However, the patient declined to provide informed consent for surgical intervention, given the absence of symptoms.

Consequently, a conservative management strategy with careful clinical and radiological surveillance was adopted. In this context, surgical intervention would be reconsidered if specific clinical or imaging changes occur, including the development of new symptoms (such as headache, visual disturbances, diplopia, or cranial nerve deficits), progressive enlargement of the lesion on follow-up imaging, the appearance of solid enhancing components, diffusion restriction suggestive of infection, or increasing mass effect on adjacent neurovascular structures.

Despite this limitation, the characteristic imaging features observed in this case strongly support the diagnosis of an extrasinusal mucocele and highlight the importance of detailed radiological evaluation when encountering atypical skull-base cystic lesions.

## Conclusion

Extrasinusal mucoceles are rare lesions that may be incidentally detected and can pose significant diagnostic challenges due to their atypical location. Despite their benign nature, accurate CT and MRI evaluation is essential for establishing the correct diagnosis, excluding mimicking entities, and guiding appropriate management and follow-up.

## Patient consent

Written informed consent was obtained from the patient.
